# Novel CD28 antagonist mPEG PV1-Fab’ mitigates experimental autoimmune uveitis by suppressing CD4+ T lymphocyte activation and IFN-γ production

**DOI:** 10.1371/journal.pone.0171822

**Published:** 2017-03-01

**Authors:** Pedro Henrique Papotto, Eliana Blini Marengo, Luiz Roberto Sardinha, Karina Inácio Carvalho, Ana Eduarda Zulim de Carvalho, Sheyla Castillo-Mendez, Carina Calixto Jank, Bernard Vanhove, Anna Carla Goldberg, Luiz Vicente Rizzo

**Affiliations:** 1 Hospital Israelita Albert Einstein, São Paulo, Brazil; 2 OSE Immunotherapeutics SA, Nantes, France; Boston University School of Medicine, UNITED STATES

## Abstract

Autoimmune Uveitis is an important chronic inflammatory disease and a leading cause of impaired vision and blindness. This ocular autoimmune disorder is mainly mediated by T CD4^+^ lymphocytes poising a T_H_1 phenotype. Costimulatory molecules are known to play an important role on T cell activation and therefore represent interesting therapeutical targets for autoimmune disorders. CD28 is the prototypical costimulatory molecule for T lymphocytes, and plays a crucial role in the initiation, and maintenance of immune responses. However, previous attempts to use this molecule in clinical practice achieved no success. Thus, we evaluated the efficacy of mPEG PV1-Fab’ (PV1), a novel selective CD28 antagonist monovalent Fab fragment in the treatment of Experimental Autoimmune Uveitis (EAU). Here, we showed that PV1 treatment decreases both average disease score and incidence of EAU. A decrease in the activation profile of both T CD4^+^ and T CD8^+^ eye-infiltrating lymphocytes was evidenced. In the periphery, T CD4^+^ cells from PV1-treated mice also showed a decrease in their activation status, with reduced expression of CD69, CD25, and PD-1 molecules. This suppression was not dependent on Treg cells, as both their frequency and absolute number were lower in PV1-treated mice. In addition, frequency of CD4^+^IFN-γ^+^ T cells was significantly lower in PV1-treated group, but not of IL-17-producing T cells. Moreover, after specific restimulation, PV1 blockade selectively blocked IFN-γ production by CD4^+^ lymphocytes Taken together, our data suggest that mPEG PV1-Fab’ acts mainly on IFN-γ-producing CD4^+^ T cells and emphasize that this specific CD28 blockade strategy is a potential specific and alternative tool for the treatment of autoimmune disorders in the eye.

## Introduction

Autoimmune uveitis is an important inflammatory disease of the eye and it is responsible for approximately 10% of visual deficit and legal blindness cases in the USA [1]. Uveitis is characterized by an inflammation of the uvea–a layer comprising the tissues between the sclera and retina; in addition, it can also be extended to adjacent tissues, such as the optic nerve and the vitreous humor [2]. Uveitis is broadly divided into infectious and non-infectious uveitis (which includes autoimmune uveitis), and can be additionally classified according to the anatomical portions of the eye affected by disease. Moreover, different autoimmune syndromes might exhibit uveitis as its clinical manifestations; that is the case, for instance, for Behçet’s disease [3], sarcoidosis [4] and reactive arthritis [5].

Despite all the variability, the immune response against ocular antigens, such as interphotoreceptor retinoid binding protein (IRBP), S-antigen or recoverin is the common feature among all forms of autoimmune uveitis [6, 7]. This response is mainly dependent on T cells; T_H_1 lymphocytes, in particular, play an important role in this autoimmune disorder, but do not seem either to initiate or sustain an immune response alone [8]. Experimental data have shown that IFN-γ knockout mice still develop autoimmune uveitis, albeit exhibiting deviant features when compared with wild-type counterparts [9]. In the past few years T_H_17 cells have also been shown to have a role in the pathogenesis of this disease [10, 11] but again, this T cell subpopulation does not seem to act alone, as demonstrated by the use of IL-17 knockout mice, which still develop eye inflammation [10].

Although a great variety of experimental models for uveitis are available [12], experimental autoimmune uveitis (EAU) is the most accepted model for human autoimmune uveitis. EAU shares key characteristics with its human equivalent disease, such as the nature of antigens, T cell involvement, and histological features. This T-cell mediated inflammatory disease is elicited by immunization of rodents with ocular antigens–mainly, IRBP or its immune-dominant epitopes–in Complete Freund’s Adjuvant (CFA), plus a *Bordetella pertussis* toxin (PTx) [13]. In mice, disease course is longer when compared to other rodent models, facilitating experimental and therapeutical handling [14]. Briefly, within four to seven days after immunization, immune cells migrate to the eye, characterizing the afferent phase of the disease. Cell migration is higher by days 13 to 15, comprising the efferent phase of the disease. By day 21, disease starts to recede, and histological signs in the eyes are clearly evident; at this phase, immune cells migrate back to draining lymph nodes (dLN) and spleen [15]. Disease severity is determined by histological analysis of the eyes, scored on a scale from 0 (no disease) to 4 (maximum disease), and based on numbers and type of lesions.

Current treatments for autoimmune uveitis are comprised of a large number of immunosuppressive drugs, such as corticosteroids, anti-metabolite drugs, T cell inhibitors and immunobiologicals [16]. However, these treatments often result in severe side effects, due to their unspecific nature. Hence, a lot of effort has been put into discovering new targets and molecules that would allow a more precise handling of the immune system.

T cells are known to play an important role in different autoimmune diseases, and are therefore interesting targets for new immunomodulatory therapies. Costimulatory pathways contribute to full activation of T lymphocytes. CD28 is the prototypic and one of the most studied co-stimulatory molecules. CD28 signaling is responsible for T cell proliferation and survival, IL-2 production, and specific T cell memory [17]. Altogether, these features encouraged researchers to consider CD28 blockade as a promising tool for immunomodulation in autoimmune diseases. Indeed, many reports showed the efficacy of anti-CD28 monoclonal antibodies in the treatment of autoimmune diseases and transplantation models [18–22]. Unfortunately, the disastrous outcomes of a phase I clinical trial with TGN1412, a superagonistic anti-CD28 antibody [23, 24] highlight the importance of developing novel antibodies with more selective specificities as a result from a deeper understanding of the mechanisms underlying this co-stimulatory blockade.

Accordingly, Abe and colleagues [25] developed mAb PV1, an anti-CD28 monoclonal antibody with no cross-reactivity against CTLA-4 or CD3 [26]and incapable of signal transduction, working as a classical antagonist. This antibody has been used in different models, with promising results. Perrin and colleagues [20] showed that mAb PV1 targets encephalomyelitogenic T cell clones and mitigates Experimental Autoimmune Encephalomyelitis. In a heart transplant model the use of PV1 IgG3 was shown to improve graft survival through modulation of the cytokine milieu [19]. Based on these results, Poirier and colleagues developed FR104, a novel humanized CD28 antagonist composed of a monovalent Fab fragment conjugated with polyethylene glycol (PEG), aiming for tolerance restoration in autoimmune conditions and in transplantation [21]. Similar to mAb PV1, FR104 failed to induce T cell responses and showed efficacy in suppressing effector T cells in a humanized Graft *versus* Host Disease model [27]. However, further knowledge of the mechanism of action of FR104 is still needed. In this study, using mPEG PV1-Fab’ (PV1), a FR104 murine analogue comprising a monovalent fragment of the mAb PV1 antibody conjugated with PEG molecules, we investigated its effect on the treatment of EAU. Here we show that PV1 decreases both disease average score and incidence, whereas decreasing overall T cell activation in the uveitic eyes and the periphery. The observed immunosuppression is not due to generation of T_reg_ cells or induction of anergy but is directed against IFN-γ production by T_H_1 cells.

## Material and methods

### Mice

B10.RIII mice were obtained from Jackson Laboratories and were maintained under specific pathogen-free conditions at Hospital Israelita Albert Einstein animal facility (CETEC), an institution certified by the Association for Assessment and Accreditation of Laboratory Animal Care. The Animal Care Committee of the Institute of Biomedical Sciences at the University of São Paulo and the Animal Care Committee of the Hospital Israelita Albert Einstein approved all the procedures utilized in this study; all procedures are in accordance to international rules of animal care as defined by the International Animal Welfare Recommendations [28].

### Antigens and reagents

Peptide SGIPYIISYLHPGNTILHVD representing residues 161–180 of IRBP was purchased from China Peptide Co., Ltd (Shanghai, China). *Bordetella pertussis* toxin (PTx) and Complete Freund Adjuvant (CFA) were purchased from Sigma-Aldrich (St. Louis, USA).

### mPEG PV1-Fab’ (PV1)

mPEG PV1-Fab’ (PV1) was provided by Dr. Bernard Vanhove (INSERM U1064, Nantes, France).Briefly, PV1 has been produced by conjugating a 40KDa polyethylene glycol moiety (Sunbright GL2-400MA; NOF Corporation, Japan) to Fab’ fragments obtained from the PV-1 antibody (ATCC HB 11944). ATCC HB 11944 is a hamster monoclonal antibody reacting against mouse CD28 (P31041; Mouse Cd28.), and not against other molecules, such as CTLA-4 (*http://web.expasy.org/cellosaurus/CVCL_8970)*.

### Induction of EAU and anti-CD28 treatment

B10.RIII mice (n = 5–10/group), male and female at 4 to 6 weeks-old, were immunized subcutaneously at the flanks with 50 μg/animal of 161–180 IRBP peptide emulsified in 200μL of complete Freund’s adjuvant (v/v) [8]. As an additional adjuvant, mice were injected intraperitoneally with 500 ng/animal of Pertussis toxin [29], in a total volume of 100 μL. Starting at day 9 post-immunization, mice were treated intraperitoneally (ip) every 4 days with 10mg/kg of PV1 until days 14 or 21, when mice were sacrificed using carbon dioxide asphyxiation and eyes, inguinal and para-aortic lymph nodes (draining lymph nodes -dLNs), and spleen were collected. In all experiments, a group of PV1-untreated mice was used as positive control of disease. Subsequently, histological analysis of the eyes, and immunophenotype and cytokine production of eye, spleen and dLNs were then assessed.

### Histological analysis and disease scoring

On day 21, eyes were collected and prepared for histological analysis as described elsewhere [30]. Disease severity affecting both untreated and PV1-treated mice was evaluated in a double-blinded fashion by examining four sections from each eye, cut at different levels. Disease was scored according to a scale from 0 (no disease) to 4 (maximum disease), in half-point increments, according to a semi-quantitative system described previously [13]. The minimal EAU score considered was characterized by inflammatory cell infiltration of the ciliary body, choroids, or retina (score = 0.5). Progressively higher scores were assigned according to the numbers and severity of findings such as vasculitis, granuloma, retinal folding, and detachment and damage to the photoreceptor layer. The individual average score of both eyes was then calculated for the final individual scores.

### Isolation of eye-infiltrating cells

Uveitic eyes were collected from at least three mice per group, either on day 14 or 21 after immunization. Both eyes were washed with phosphate buffer-saline (PBS) plus 2% fetal bovine serum (FBS) and were carefully mashed between two sterile nylon membranes with 2mL of PBS plus 2% FBS in a Petri dish; cells were then centrifuged. The supernatant was discarded and the cell pellet resuspended in 1mL of Lysing Buffer (BD Biosciences, San Diego, USA) for 3 minutes at 37°C. Subsequently, 10mL of PBS plus 2% FBS was added and cells were again centrifuged. The supernatant was discarded and the cell pellet was resuspended in 1mL of PBS plus 2% FBS for posterior cell counting and immunophenotyping as described below.

### Eye, spleen and dLNs immunophenotype by flow cytometry

Infiltrating cells from the lymph nodes draining the immunization site (inguinal and para-aortic lymph nodes), spleen, and eyes were isolated and labeled with the following antibodies: anti-CD4, anti-CD45, anti-CD62L, anti-CD44, anti-Tim3, anti-CD69, anti-NKG2D, anti-CD8, anti-CD3, anti-PD-1, anti-BTLA and anti-CTLA-4 (BioLegend Inc., San Diego, USA); anti-CD25 and anti-Foxp3 (BD Biosciences, San Diego, USA). Intranuclear Foxp3 staining was performed using the Foxp3 fixation/permeabilization buffers from BD Biosciences (San Diego, USA).

For intracellular detection of IFN-γ and IL-17, dLN cells were cultured overnight with 100ng/mL of phorbol myristate acetate (PMA) and 500ng/mL of ionomycin (Sigma-Aldrich, St. Louis, USA) in the presence of Golgi Plug (brefeldin A) at the recommended concentrations (BD Biosciences, San Diego, USA). Non-stimulated cells were used as controls. PV1-treated and untreated mice cells were then harvested and labeled for anti-CD3, anti-CD4 and anti-CD8 (BioLegend Inc., San Diego, USA). Cells were then fixed and permeabilized using Cytofix/Cytoperm reagents from BD Biosciences (San Diego, USA). For intracellular staining of cytokines the following antibodies were used: anti- IFN-γ, anti-IL-17 and anti-IL-2 (BD Biosciences, San Diego, USA). Cells were acquired using a LSR Fortessa (BD Biosciences, San Diego, USA) and analyzed with FlowJo (TreeStar Inc., Ashland, USA) and Pestle (version 1.7)/ SPICE (version 5.3; M. Roederer, Vaccine Research Center, National Institute of Allergy and Infectious Diseases, National Institutes of Health) as described elsewhere [31].

### Statistical analysis

Experiments were repeated at least three times. Experimental groups were typically composed of five mice. Figures show data combined from three independent experiments, unless stated otherwise. For statistical analysis of EAU scores, each mouse (average of both eyes) was considered as one statistical event. Groups were analyzed using two-tailed Mann-Whitney tests and statistical significance was set for *p*<0.05. All the analyses were performed using GraphPad Prism 4.0 software (GraphPad Software Inc., La Jolla, USA).

## Results

### PV1 treatment decreases EAU scores

**Fig 1:**
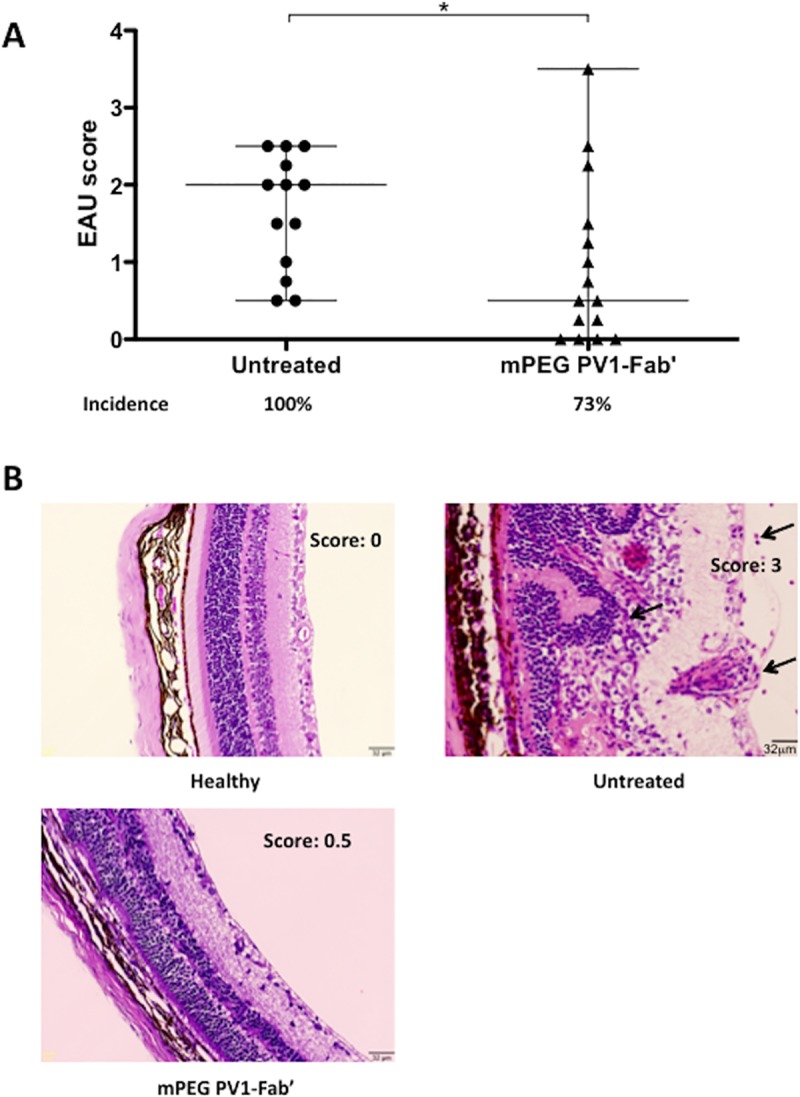
PV1 treatment ameliorates EAU. B10.RIII mice were immunized with 50 μg/animal of 161–180 IRBP in CFA, plus 500 ng/animal of PTx boost. Starting on day 9, mice were treated ip every 4 days with the CD28 antagonist PV1 (10mg/Kg), or left untreated. On day 21 mice were sacrificed and eyes were collected for histology analysis. (A) EAU scores were assigned in a 0 to 4 scale, in a double-blinded fashion. (B) Representative H&E staining of eyes (Magnification 200x) from healthy mice, PV1-treated and untreated group. Histological findings such as inflammatory infiltrate, vasculitis and granuloma are indicated by black arrows. Data combined from 4 independent experiments, 5–10 mice per group. Median and range are depicted in the scattered plot. *, p<0.05, two-tailed Mann-Whitney test.

To evaluate the effect of PV1 on the progression of EAU, mice were immunized with 161–180 IRBP peptide and, on day 9 post-immunization, were either treated with PV1 or left untreated. At this time point, disease is in the end of its afferent phase and inflammatory T lymphocytes are already found in the eyes. Mice were treated with PV1 every four days and were sacrificed on day 21 to score EAU disease by histological analysis. PV1 treatment significantly (p<0.05, two-tailed Mann-Whitney test) decreased both average EAU score (0.95±1.07) and incidence (69%) when compared with the untreated group (1.65±0.75 and 100%, respectively) (Fig 1A). Accordingly, the lower score was characterized by a less pronounced inflammatory response with lower incidence of vasculitis, granuloma formation, and retinal folding when compared with untreated animals (Fig 1B). No apparent adverse effects, such as weight loss, lethargy or death were observed in PV1-treated and controls mice.

### Diminished EAU severity is accompanied by a decrease in the activation profile of eye-infiltrating T lymphocytes

**Fig 2:**
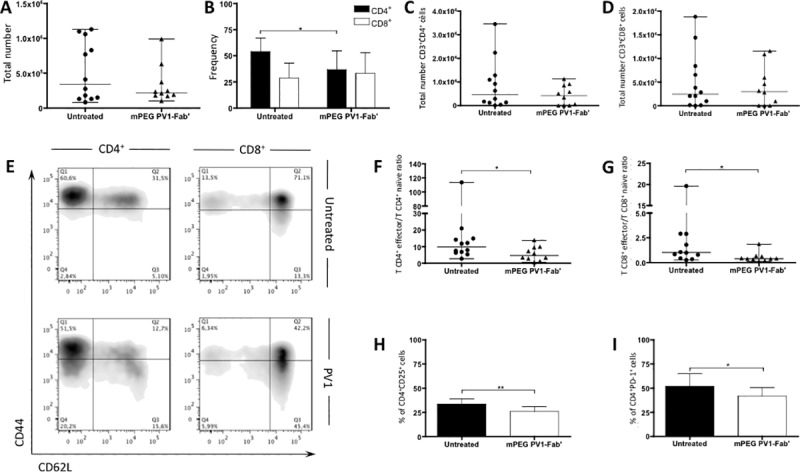
PV1-treated mice exhibited less activated eye-infiltrating lymphocytes. B10.RIII mice were immunized with 50 μg/animal of 161–180 IRBP in CFA, plus 500 ng/animal of PTx boost. Starting on day 9, mice were treated every 4 days with CD28 antagonist, PV1 (10mg/Kg; ip), or left untreated. On day 14 mice were sacrificed and eyes were collected for immunophenotyping of eye-infiltrating leukocytes. (A) Total count of eye-infiltrating leukocytes. (B) Frequency of CD4^+^ and CD8^+^ T lymphocytes infiltrating the eyes of B10.RIII mice. (C) Total number of CD4^+^ T lymphocytes and (D) CD8^+^ T lymphocytes. (E) Representative plots display CD44 and CD62L expression by CD4^+^ and CD8^+^ cells. (F) T_effector_/T_naïve_ ratio (as defined by CD44 and CD62L expression) for CD4^+^ and (G) CD8^+^ T lymphocytes. (H) Frequency of CD4^+^CD25^+^ T cells in uveitic eyes. (I) Frequency of CD4^+^PD-1^+^ T cells uveitic eyes. Data combined from three independent experiments; 5–10 mice per group. In (A), (C), (D), (F) and (G), median and range are depicted. In (B), (H) and (I) Mean ± SD are depicted.*, p<0.05, two-tailed Mann-Whitney test.

Since PV1 decreased EAU, the effects of PV1 on T cells infiltrating the eyes of B10.RIII mice were investigated. On day 14, when the disease is already established and the inflammatory infiltrate is at its peak, eyes were collected for immunophenotyping of infiltrating cells. Both untreated and PV1-treated mice showed similar number of eye-infiltrating cells (Fig 2A), and of the CD4^+^ and CD8+ T cell populations (Fig 2C and 2D) as well. However, it was observed a lower frequency of CD4^+^ T cells in the PV1-treated group (Fig 2B). Of note, no differences between PV1-treated mice and controls were found for B lymphocyte and NK cell frequencies (S1A Fig).

As PV1 treatment did not seem to interfere with T cell migration to the eyes (Fig 2A), we next sought to investigate the activation profile (assessed by the expression of CD44 and CD62L) of eye-infiltrating T lymphocytes. PV1-treated mice showed a lower T effector/T naïve ratio for both CD4^+^ (Fig 2E and 2F) and CD8^+^ (Fig 2E and 2G) cells when compared to the controls. Additionally, CD25 and PD-1 in CD4^+^ and CD8^+^ cells, both markers of T cell activation, were decreased in PV1-treated animals (Fig 2H and 2I).

Taken together, the results indicate that the CD28 blockade achieved with PV1 dampens the activation of eye-infiltrating T cells rather than interfering with their homing to the eye.

### CD28 blockade with PV1 decreases overall T cell activation in periphery

**Fig 3:**
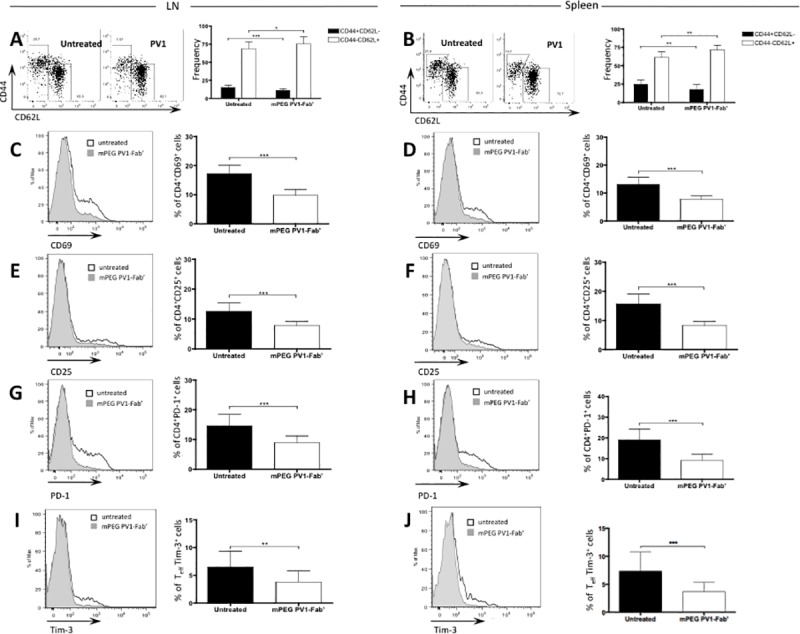
PV1-treated mice exhibited a decrease in overall activation of T lymphocytes. B10.RIII mice were immunized with 50 μg/animal of 161–180 IRBP in CFA, plus 500 ng/animal of PTx boost. Starting on day 9, mice were treated every 4 days with CD28 antagonist, PV1 (10mg/Kg; ip), or left untreated. On day 14 mice were sacrificed and spleen and dLN were collected for immunophenotyping. (A) Representative plot for CD44 and CD62L expression in T CD4^+^ lymphocytes, and frequencies of CD44^+^CD62L^-^ (effector) and CD44^-^CD62L^+^ (naïve) CD4^+^ T lymphocytes in dLN and (B) spleen. (C) Representative plot for expression of CD69 in CD4^+^ lymphocytes and frequency of CD4^+^CD69^+^ T cells in dLN and (D) spleen. (E) Representative plot for expression of CD25 in CD4^+^ lymphocytes and frequency of CD4^+^CD25^+^ T cells in dLN and (F) spleen. (G) Representative plot for expression of PD-1 in CD4^+^ lymphocytes and frequency of CD4^+^PD-1^+^ T cells in dLN and (H) spleen. Data combined from three independent experiments; 5 mice per group. Representative plot for expression of Tim-3 in CD4^+^CD44^+^CD62L^-^ lymphocytes and frequency of CD4^+^CD44^+^CD62L^-^Tim-3^+^ T cells in (I) dLN and (J) spleen. Data combined from three independent experiments; 5 mice per group. Mean ± SD are depicted.*, p<0.05; **, p<0.01; ***, p<0.0001, two-tailed Mann-Whitney test.

To evaluate if our findings were confined to the inflammatory environment of uveitic eyes, dLN and spleen were collected on day 14 for analysis of T lymphocyte activation profile. Again, total cell count was similar in both dLN and spleen in both groups (S2A Fig). Moreover, similar frequencies of CD4^+^, CD8^+^, CD19^+^ and NK cells were found in spleen and dLN from both untreated and PV1-treated group (S2B Fig). However, similar to findings in the eye, CD4^+^ T cells from PV1-treated mice exhibited lower frequencies of effector cells evaluated here as CD44^+^CD62L^-^ cells and higher frequencies of naïve cells (CD44^-^CD62L^+^ cells), in both dLN and spleen (Fig 3A and 3B). Furthermore, other activation markers, expressed at different time points of a T cell response were also altered in PV1-treated mice. The frequency of CD4^+^CD69^+^ (Fig 3C and 3D), CD4^+^CD25^+^ (Fig 3E and 3F), CD4^+^PD-1^+^ (Fig 3G and 3H), and CD4^+^Tim-3^+^ cells (Fig 3I and 3J) in PV1-treated animals was lower than in their untreated counterparts, in both spleen and dLN. Likewise, CD69, CD25 and PD-1 mean fluorescence intensity (MFI) were lower in CD4^+^ cells from spleen and dLN from PV1-treated mice, when compared to their untreated counterparts (S3 Fig). In addition, though there were no differences in naïve and effector CD8^+^ T cell frequencies (S4A and S4B Fig), CD8^+^CD69^+^ (S4C and S4D Fig) and CD8^+^PD-1^+^ (S4E and S4F Fig) T cells were lower in both spleen and dLN of PV1-treated mice. Therefore, we conclude that PV1 decreases overall T cell activation in the periphery with decreased expression of different costimulatory and activation molecules.

### PV1 treatment decreases the T_reg_ population in peripheral lymphoid organs

**Fig 4:**
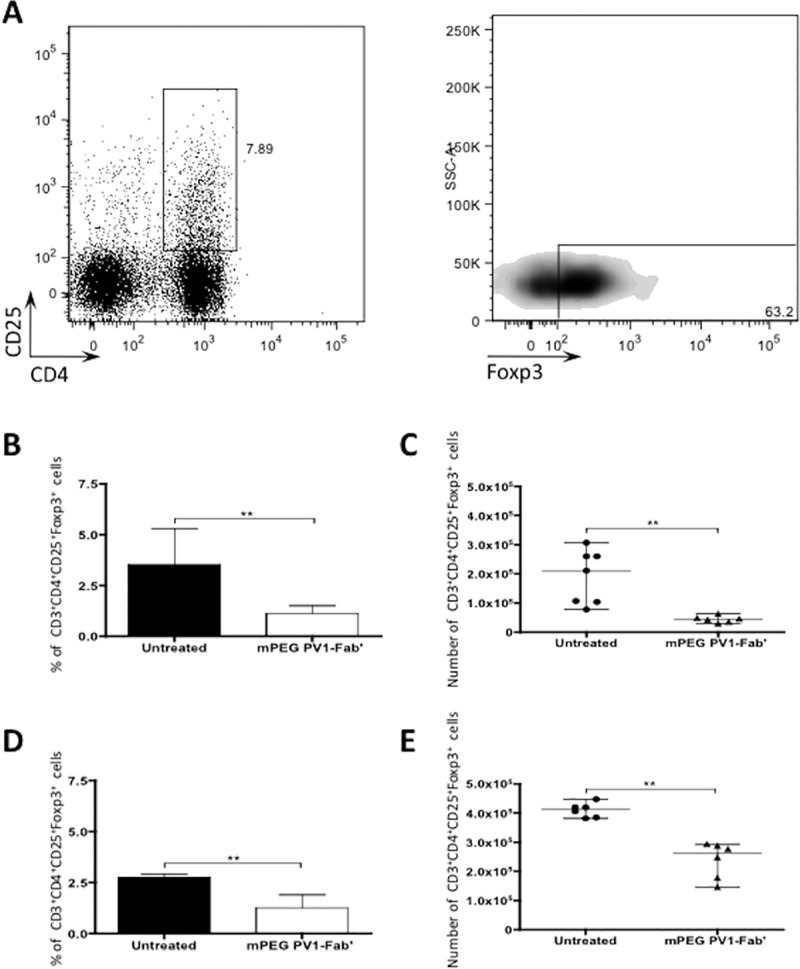
mPEG PV1-Fab’ treatment decreases T_reg_ population. Female B10.RIII mice were immunized with 50 μg/animal of 161–180 IRBP in CFA, plus 500 ng/animal of PTx boost. Starting on day 9, mice were treated every 4 days with CD28 antagonist, PV1 (10mg/Kg; ip), or left untreated. On day 14 mice were sacrificed and dLN were collected for immunophenotyping of regulatory T cells. (A) Representative plot showing gate strategy for defining T_reg_ population. Foxp3 expression was defined by using a Fluorescence Minus One (FMO) control. (B) Frequency and (C) total number of CD3^+^CD4^+^CD25^+^Foxp3^+^ cells in dLN of B10.RIII mice. (D) Frequency and (E) total numbers of CD3^+^CD4^+^CD25^+^Foxp3^+^ cells in spleen of B10.RIII mice. Data are representative of three independent experiments; 5-mice per group. Median and range are depicted. **, p<0.01, two-tailed Mann-Whitney test.

Regulatory T cells are responsible for controlling T cell activation and effector responses and have been shown to reduce the severity of several autoimmune disorders [32]. In addition to well-known mechanisms, T_reg_ cell generation can also occur due to incomplete activation of T lymphocytes [33, 34]. Consequently, it was conceivable that CD28 blockade by PV1 might increase T_reg_ frequency and mitigate progression of autoimmunity in our uveitis model. Therefore, we evaluated Treg lymphocytes population in dLN and spleen (Fig 4A). Surprisingly, PV1 treatment led to a decrease in both frequency and absolute numbers of T_reg_ in both dLN (Fig 4B and 4C) and spleen (Fig 4D and 4E). Taken together, these results showed that PV1 exert a blocking effect on T_reg_ generation and that the decrease in disease severity seemingly occurs due to a direct effect upon the effector T lymphocytes.

### PV1 treatment decreases T_H_1 cell population, but has no effect on T_H_17 cells

**Fig 5:**
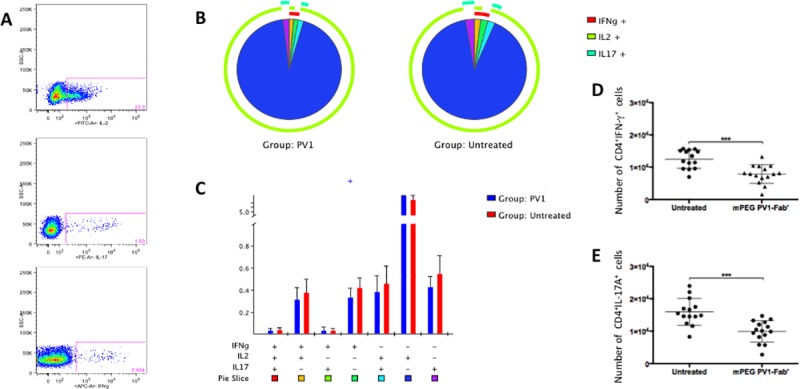
mPEG PV1-Fab’ dampens IFN-γ production by CD4^+^ T lymphocytes. B10.RIII mice were immunized with 50 μg/animal of 161–180 IRBP in CFA, plus 500 ng/animal of PTx boost. Starting on day 9, mice were treated every 4 days with CD28 antagonist, PV1 (10mg/Kg; ip), or left untreated. On day 14 mice were sacrificed and dLN were collected for immunophenotyping and evaluation of cytokine production. For the intracellular staining of IFN-γ and IL-17 cells were collected from dLN (3 mice/group), plated at 1x10^6^ cells/well concentration and stimulated overnight with 100 ng/mL of PMA and 500 ng/mL of ionomycin, plus GolgiPlug at manufacturer’s recommended concentrations. (A) Representative plots show IFN-γ, IL-17 and IL-2 production by CD3^+^CD4^+^ cells. (B) Pie charts (C) and absolute frequency of CD4^+^IFN-γ^+^ cells. In brief, each subpopulation depicted in the bar graph is also depicted in the pie chart as pie slices, following the same color code. Overall IFN-γ, IL-17 and IL-2 production is displayed as the outer arcs in the pie charts. (D) Total numbers of CD4^+^IFN-γ^+^ cells. Mean ± SD are depicted in (C) and (D). **, p<0.01; ***, p<0.0001, two-tailed Mann-Whitney test.

EAU pathogenesis is greatly dependent on T_H_1 lymphocytes [8] but T_H_17 cells also were shown to participate in the progression of disease [11]. As PV1 effects did not seem to be mediated by T_reg_ cells in EAU, we next explored the effects of PV1 treatment on IFN-γ, IL-17, and IL-2 production by CD4^+^ T lymphocytes (Fig 5A). In PV1-treated mice CD4^+^IFN-γ^+^ T cells (Fig 5B, 5C and 5D) were significantly reduced both in number and frequency in dLN. In contrast, there were no changes in the frequencies of CD4^+^IL-17^+^ (Fig 5B and 5C), CD4^+^IL-2^+^ T cells (Fig 5B and 5C), or in IFN-γ-, IL-2-, and IL-17-producing T CD8+ cells (S5A, S5B and S5C Fig). However, a decrease in the numbers of CD4^+^IL-17^+^ was also observed (Fig 5E).

These results suggest that PV1 acts mainly on the IFN-γ production by CD4+ T cells.

### CD28 blockade with PV1 prevents T_H_1 cell expansion after antigen re-encounter

**Fig 6:**
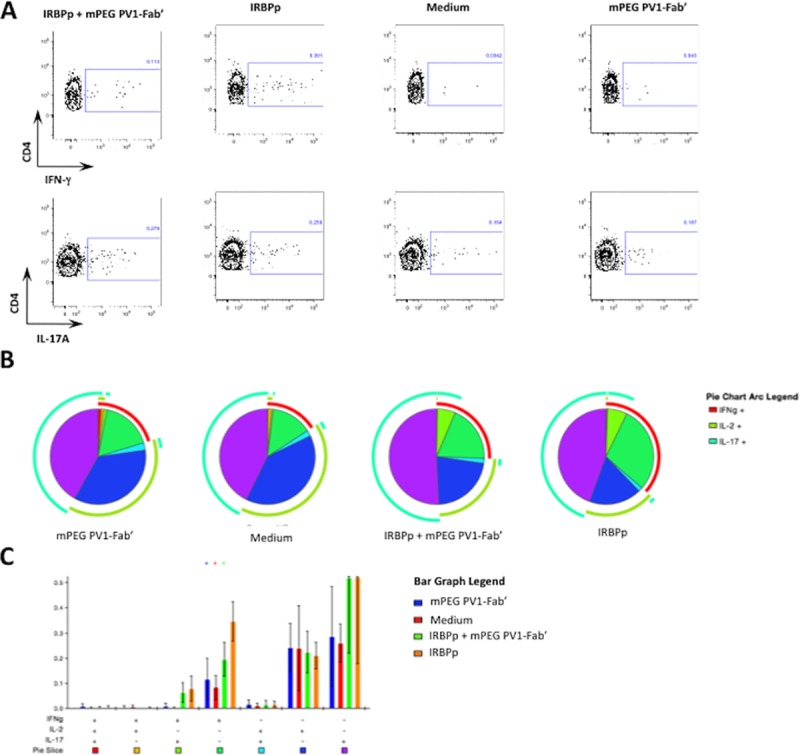
CD28 blockade with mPEG PV1-Fab’ prevents T_H_1 cell expansion after antigen re-encounter. B10.RIII mice were immunized with 50 μg/animal of 161–180 IRBP in CFA. On day 7 mice were sacrificed and dLN were collected for evaluation cytokine production. For the intracellular staining of IFN-γ, IL-17 and IL-2 cells were collected from dLN, pre-incubated or not with 10μg/mL of PV1, and plated at 1x10^6^ cells/well concentration and stimulated or not for 48h with IRBP 161–180 peptide (IRBPp). (A) Pie charts showing IFN-γ, IL-17 and IL-2 production by CD3^+^CD4^+^ cells. (B) Absolute frequency of CD4^+^IFN-γ^+^ cells. In brief, each subpopulation depicted in the bar graph is also depicted in the pie chart as pie slices, following the same color code. Overall IFN-γ, IL-17 and IL-2 production is displayed as the outer arcs in the pie charts. Data combined from three independent experiments. Mean ± SD are depicted in (B)**, p<0.01; ***, p<0.0001, two-tailed Mann-Whitney test.

Finally, T cells primed for ocular antigens in dLN migrate to the eyes, where they exert their effector functions [8] leading to disease. The decrease in T_H_1 lymphocytes population observed in PV1-treated mice could be due to a direct effect of CD28 blockade on these cells, by blocking survival signals and effector functions. With the purpose of mimicking the events following T cell priming in the periphery, *in vitro* assays were performed. Cells were collected on day 7 post-immunization from dLN of EAU mice, incubated with PV1 and stimulated with the 161–180 IRBP peptide (mimicking the antigen re-encounter); these data were compared to cells without incubation with PV1. CD28 blockade with PV1 led to a decrease in CD4^+^IFN-γ^+^ T cell frequency, when compared to untreated cells (Fig 6A, 6B and 6C). T_H_17 cell population and IL-2 production were not affected by PV1 treatment (Fig 6A, 6B and 6C), confirming that PV1 acts on primed T lymphocytes, therefore leading to lower IFN-γ production.

## Discussion

In this study we show that the selective blockade of CD28 using a monovalent Fab fragment is effective in the treatment of experimental autoimmune uveitis acting directly on effector T cells, specifically dampening IFN-γ production with no induction of anergy or enhancement of T_reg_ cell activity. These results are in line with previous data where complete anti-CD28 antibodies were employed [19, 20, 22], confirming this strategy as a valid therapeutic option for autoimmune uveitis. Moreover, PV1 human homologue, FR104, was shown to be effective in preventing renal allograft rejection ([35]), and in the treatment of skin inflammation [36, 37] and experimental autoimmune encephalomyelitis [38] in non-human primates. Nonetheless, a careful evaluation is opportune, to ensure no unexpected events will arise due to unforeseen interactions by the effector Fc portions of the antibody or cross-reactivity with CTLA-4 [27, 33].

Different immunotherapies promote their suppressive effects by sequestering effector lymphocytes to the peripheral lymphoid organs and preventing migration of the pathogenic cells to inflamed tissues. That is the case for FTY720 [39] and the monoclonal antibody natalizumab [40, 41]. CD28 is known to control circulation of auto-reactive T lymphocytes through IL-2–inducible Tec kinase (ITK) signaling [42]. However, the lack of differences between untreated and PV1-treated groups regarding the total number of eye-infiltrating and dLN cells discards this explanation. Instead, the observed decrease in the ratio of effector/naïve eye-infiltrating T cells and the lower expression of CD25 and PD-1 in PV1-treated animals raises the possibility that PV1 is hindering the activation of effector T lymphocytes. In line with these findings, the decrease in overall activation status of lymphocytes found in peripheral lymphoid organs from mice treated with PV1, showed that CD28 blockade prevented the full activation of T CD4^+^ and T CD8^+^ lymphocytes. In accordance to the two-signal theory of T cell activation, CD28 engagement has been shown to promote expression of additional costimulatory and activation molecules and inhibit the expression of co-inhibitory molecules [43]. In particular, upregulation of CD25 is a key feature in CD28 signaling through the IL-2 pathway [44]. The CD28-IL-2 axis also has an important role on the induction and activity of PD-1, as IL-2 produced after CD28 engagement is responsible for the inhibition of PD-1 expression [45]. Thus, we expected that CD28 blockade by PV1 would generate inhibitory signals mediated by upregulation of coinhibitory molecules [33]. Our results, however, showed otherwise. Both costimulatory and coinhibitory molecules were downregulated in PV1-treated mice. As CD28 is constitutively expressed on T cells and one of the first costimulatory signals triggered upon T cell activation, blocking this pathway would stop the ensuing expression of surface molecules such as CD69, CD25, and PD-1, which would, in turn, direct immune responses to an arrested pattern.

Arrested T cell activity is achieved by several mechanisms, a major one being by way of regulatory T lymphocytes and another one by IL-2 deprivation [46], which dampens proliferation and survival of T lymphocytes causing anergy.

T_reg_ lymphocytes are generated in the periphery in order to build and maintain peripheral tolerance and to control responses to external antigens [32]. Accordingly, incomplete activation of T cells, with lack of costimulatory signals is thought to result in either anergy or T_reg_ generation [34]. In fact, the monovalent anti-CD28 antibody FR104 was shown to enhance T_reg_ function [33] leading us to hypothesize that CD28 blockade with PV1 antibody would induce T_reg_ lymphocytes, blocking T cell activation and decreasing EAU severity. Surprisingly, PV1-treated mice exhibited lower frequencies and absolute numbers of T_reg_ in peripheral lymphoid organs, indicating a detrimental effect of PV1 on this T lymphocyte subpopulation. Although unexpected, these results are supported by previous findings that suggest an important role for CD28 in maintaining regulatory T cell homeostasis [47–49]. Still, these data need to be further explored, as this apparent T_reg_ depletion could be actually the result of T_reg_ migration to uveitic eyes and/or the enhancement of T_reg_ activity, as previously observed in a kidney transplantation model using the CD28 selective blockade strategy [33].

The blockade of CD28 signaling pathway can also induce anergy in T lymphocytes, which could be, in turn, responsible for the immune suppression observed in PV1-treated mice. In experimental models of heart transplantion and autoimmune encephalomyelitis use of PV1-IgG3 or mAb-PV1 resulted in a decrease in IL-2 production of treated animals [19, 20] suggesting anergy is one of the mechanisms of action of this monovalent antibody. However, in the present study no differences between PV1-treated and untreated groups were found regarding IL-2 production, either after specific or unspecific stimulatory conditions.

The decrease of EAU severity observed in PV1-treated mice apparently is T_reg_ independent and is not achieved through anergy induction. Thus, the decrease of IFN-γ- or IL-17-producing cells and disruption of T helper lymphocyte effector functions could explain the effect of PV1 on EAU pathogenesis [50–54]. Costimulatory molecules can induce different cytokines leading to differential polarization of T cells [55, 56] and blockade of B7.1 and B7.2 results in decreased IFN-γ production and EAU severity [22]. In addition, in a heart transplant model, PV1-IgG3 treatment led to a reduction of IFN-γ mRNA levels, whilst enhancing graft survival [19]. Moreover, Poirier and colleagues [36] observed that FR104 pre-treated leukocytes produced less IFN-γ after antigen restimulation. These findings are in line with our results, as the observed dampening on IFN-γ production and decreased frequencies of T_H_1 cells explains the effectiveness of PV1 on mitigating EAU, whilst the remainder of IL-17-producing T lymphocytes would explain the residual disease found in some mice. Although we cannot exclude that PV1 acts also in the reactivation of T_H_17 cells, our *in vitro* findings, using specific antigen restimulation of dLN cells pre-treated with PV1, point to a T_H_1 directed mechanism, as we did not find differences in the T_H_17 populations after restimulation. Thus, the decrease in T_H_17 cell numbers observed *in vivo* could be due to the overall suppression observed in CD4^+^ T lymphocytes. Also, it is important to note that Ville and colleagues [35] also found no differences in T_H_17 cells after FR104 treatment in a renal allograft model. As the time window for PV1 treatment is less than 2 weeks in the present study, and usually in the EAU mouse model it takes at least 6 weeks to the lesions decrease in number and severity [13] it seems unlikely that PV1 blockade acts promoting the recovery of the ocular lesions. Altogether, our data suggests that PV1 blockade acts by suppressing effector T cell responses in general, but mainly in antigen-specific T_H_1 lymphocytes rendering these cells unable to exert their effector functions upon antigen re-encounter in the target organ, similar to what was found after FR104 treatment in non-human primate models of skin inflammation [36] and autoimmunity [38].

## Conclusion

Here we show that specific CD28 blockade, is a promising strategy for treating autoimmune uveitis, and that PV1 is a useful tool for dissecting the cellular events involved in this phenomenon. In an EAU mice model, PV1 suppressive effects were directed towards T_H_1 lymphocytes. Improvement of the disease occurred without the desired enhancement of regulatory T cell function raising interesting issues on the roles of costimulatory molecules in the presence of a targeted CD28 blockade. In particular, the understanding of the effects of CD28 signaling on T_reg_ is crucial, as most of modern immunomodulatory strategies aim to regulate this T lymphocyte subpopulation activity. Moreover, knowing the precise mechanisms of action of mPEG PV1-Fab’ would help to improve future advances in this field, and specific and alternative treatments for autoimmune uveitis.

## Supporting information

S1 Fig Immunophenotyping of eye-infiltrating leukocytes. Female B10.RIII mice were immunized with 50 μg/animal of 161–180 IRBP in CFA, plus 500 ng/animal of PTx boost. Starting on day 9, mice were treated ip every 4 days with the CD28 antagonist PV1 (10mg/Kg), or left untreated. On days 14 or 21 mice were sacrificed and eyes were collected for immunophenotyping. (A) Frequencies of leukocyte subpopulations infiltrating uveitic eyes of B10.RIII mice on day 14 post-immunization. (B) T_effector_/T_naïve_ ratio (as defined by CD44 and CD62L expression) for CD4^+^ and (C) CD8^+^ T lymphocytes on day 21 post-immunization. (A) Data representative from at least two independent experiments; (B and C) Data combined from two independent experiments
(TIFF)

S2 Fig Immunophenotyping of dLN leukocytes. Female B10.RIII mice were immunized with 50 μg/animal of 161–180 IRBP in CFA, plus 500 ng/animal of PTx boost. Starting on day 9, mice were treated ip every 4 days with PV1 (10mg/Kg) or left untreated. On day 14 mice were sacrificed and dLN were collected for immunophenotyping. (A) Total count of dLN leukocytes. (B) Frequencies of leukocyte subpopulations in dLN. (A) Data representative from at least two independent experiments. (B) Data combined from two independent experiments.
(TIFF)

S3 Fig PV1-treated mice exhibited a decrease in overall activation of T lymphocytes. Female B10.RIII mice were immunized with 50 μg/animal of 161–180 IRBP in CFA, plus 500 ng/animal of PTx boost. Starting on day 9, mice were treated every 4 days with CD28 antagonist, PV1 (10mg/Kg; ip), or left untreated. On day 14 mice were sacrificed and spleen and dLN were collected for immunophenotyping. CD25 MFI on CD4^+^ T cells in dLN and (A) spleen (B). PD-1 MFI on CD4^+^ T cells in dLN and (C) spleen (D). CD69 MFI on CD4^+^ T cells in dLN (E) and spleen (F). Tim-3 MFI on CD4^+^CD44^+^CD62L^-^ in dLN (G) and spleen (H). Data combined from three independent experiments; 5 mice per group per experiment. Mean ± SD are depicted.*, p<0.05; ****, p<0.0001, two-tailed Mann-Whitney test.
(TIFF)

S4 Fig PV1-treated mice exhibited a decrease in the expression of activation molecules in T CD8^+^ lymphocytes. Female B10.RIII mice were immunized with 50 μg/animal of 161–180 IRBP in CFA, plus 500 ng/animal of PTx boost. Starting on day 9, mice were treated ip every 4 days with PV1 (10mg/Kg) or left untreated. On day 14 mice were sacrificed and spleen and dLN were collected for immunophenotyping. (A) Frequency of CD44^+^CD62L^-^ (effector) and CD44^-^CD62L^+^ (naïve) CD8^+^ T lymphocytes in dLN and (B) spleen. (C) Frequency of CD8^+^CD69^+^ T cells in dLN and (D) spleen. (E) Frequency of CD8^+^PD-1^+^ T cells in dLN and (F) spleen. Data combined from three independent experiments; **, p<0.01; ***, p<0.0001, two-tailed Mann-Whitney test.
(TIFF)

S5 Fig mPEG PV1-Fab’ has no effect on cytokine production by CD8^+^ lymphocytes. Female B10.RIII mice were immunized with 50 μg/animal of 161–180 IRBP in CFA, plus 500 ng/animal of PTx boost. Starting on day 9, mice were treated ip every 4 days with PV1 (10mg/Kg) or left untreated. On day 14 mice were sacrificed and dLN were collected for immunophenotyping and evaluation of cytokine production. For the intracellular staining of IFN-γ, IL-2 and IL-17 cells were collected from dLN, plated at 1x10^6^ cells/well concentration and stimulated overnight with 100 ng/mL of PMA and 500 ng/mL of ionomycin, plus GolgiPlug at manufacturer’s recommended concentrations. (A) Representative plots showing IFN-γ, IL-17 and IL-2 production by CD3^+^CD8^+^ cells. (B) Pie charts (C) and absolute frequency of CD8^+^IFN-γ^+^ cells.
(TIFF)
