# Supplementary figures and images for: Novel CD28 antagonist mPEG PV1-Fab’ mitigates experimental autoimmune uveitis by suppressing CD4+ T lymphocyte activation and IFN-γ production
Source: PLoS One. 2017 Mar 1;12(3):e0171822. doi: 10.1371/journal.pone.0171822 (PMC5331984; doi:10.1371/journal.pone.0171822)

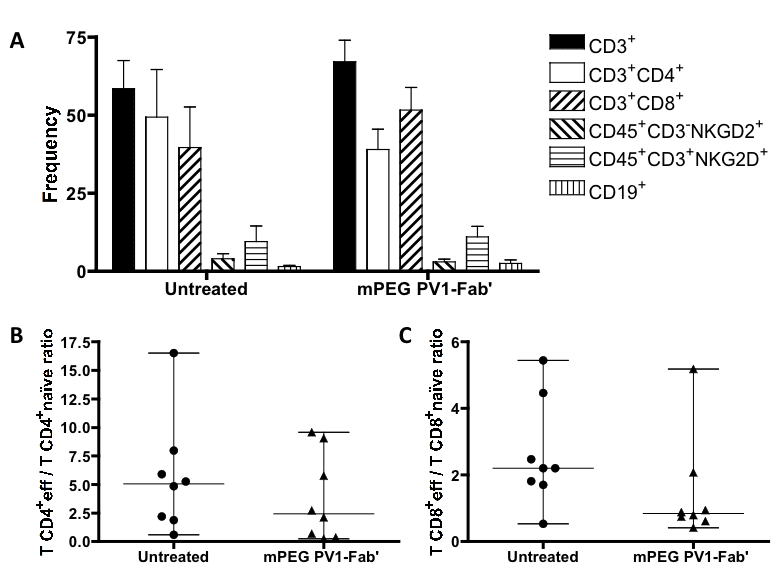

Supplement: S1 Fig — Female B10.RIII mice were immunized with 50 μg/animal of 161–180 IRBP in CFA, plus 500 ng/animal of PTx boost. Starting on day 9, mice were treated ip every 4 days with the CD28 antagonist PV1 (10mg/Kg), or left untreated. On days 14 or 21 mice were sacrificed and eyes were collected for immunophenotyping. (A) Frequencies of leukocyte subpopulations infiltrating uveitic eyes of B10.RIII mice on day 14 post-immunization. (B) Teffector/Tnaïve ratio (as defined by CD44 and CD62L expression) for CD4+ and (C) CD8+ T lymphocytes on day 21 post-immunization. (A) Data representative from at least two independent experiments; (B and C) Data combined from two independent experiments (TIFF) [file pone.0171822.s001.tiff]

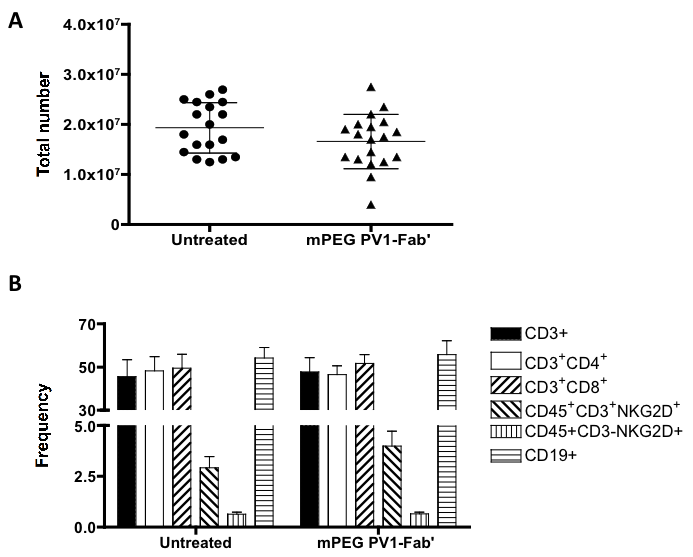

Supplement: S2 Fig — Female B10.RIII mice were immunized with 50 μg/animal of 161–180 IRBP in CFA, plus 500 ng/animal of PTx boost. Starting on day 9, mice were treated ip every 4 days with PV1 (10mg/Kg) or left untreated. On day 14 mice were sacrificed and dLN were collected for immunophenotyping. (A) Total count of dLN leukocytes. (B) Frequencies of leukocyte subpopulations in dLN. (A) Data representative from at least two independent experiments. (B) Data combined from two independent experiments. (TIFF) [file pone.0171822.s002.tiff]

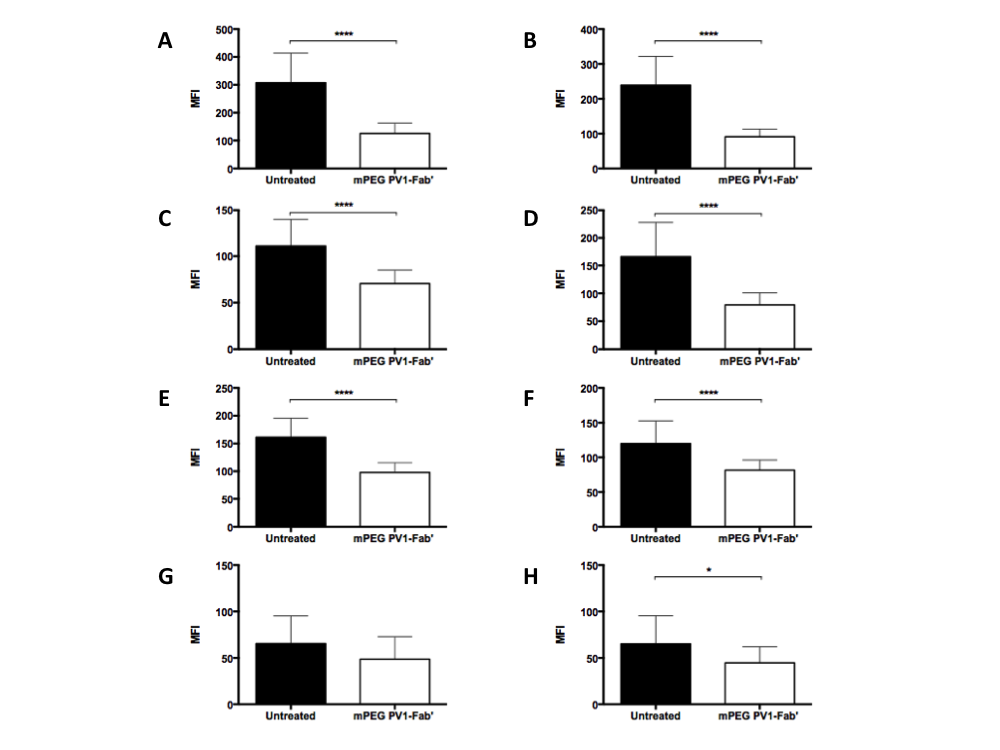

Supplement: S3 Fig — Female B10.RIII mice were immunized with 50 μg/animal of 161–180 IRBP in CFA, plus 500 ng/animal of PTx boost. Starting on day 9, mice were treated every 4 days with CD28 antagonist, PV1 (10mg/Kg; ip), or left untreated. On day 14 mice were sacrificed and spleen and dLN were collected for immunophenotyping. CD25 MFI on CD4+ T cells in dLN and (A) spleen (B). PD-1 MFI on CD4+ T cells in dLN and (C) spleen (D). CD69 MFI on CD4+ T cells in dLN (E) and spleen (F). Tim-3 MFI on CD4+CD44+CD62L- in dLN (G) and spleen (H). Data combined from three independent experiments; 5 mice per group per experiment. Mean ± SD are depicted.*, p<0.05; ****, p<0.0001, two-tailed Mann-Whitney test. (TIFF) [file pone.0171822.s003.tiff]

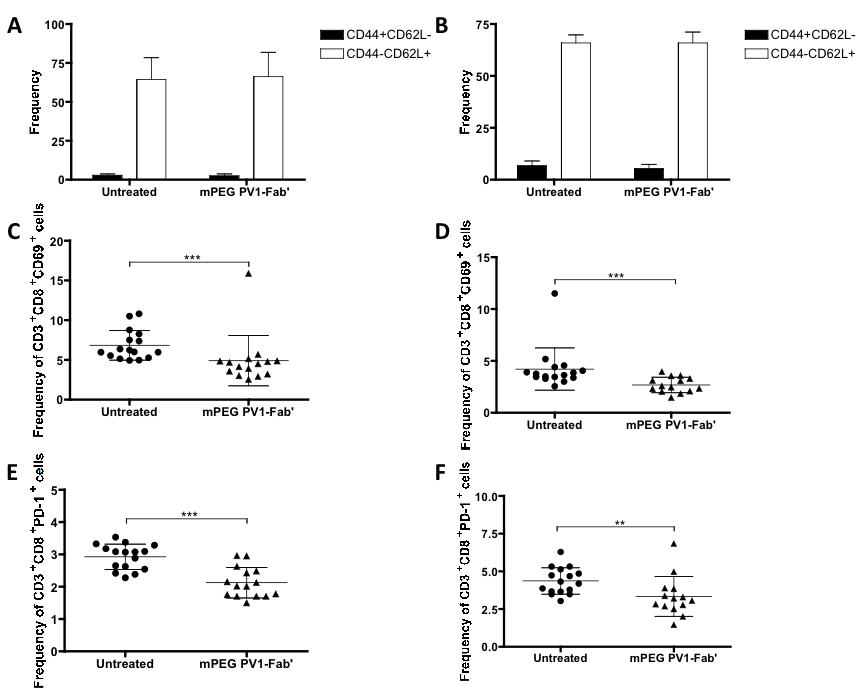

Supplement: S4 Fig — Female B10.RIII mice were immunized with 50 μg/animal of 161–180 IRBP in CFA, plus 500 ng/animal of PTx boost. Starting on day 9, mice were treated ip every 4 days with PV1 (10mg/Kg) or left untreated. On day 14 mice were sacrificed and spleen and dLN were collected for immunophenotyping. (A) Frequency of CD44+CD62L- (effector) and CD44-CD62L+ (naïve) CD8+ T lymphocytes in dLN and (B) spleen. (C) Frequency of CD8+CD69+ T cells in dLN and (D) spleen. (E) Frequency of CD8+PD-1+ T cells in dLN and (F) spleen. Data combined from three independent experiments; **, p<0.01; ***, p<0.0001, two-tailed Mann-Whitney test. (TIFF) [file pone.0171822.s004.tiff]

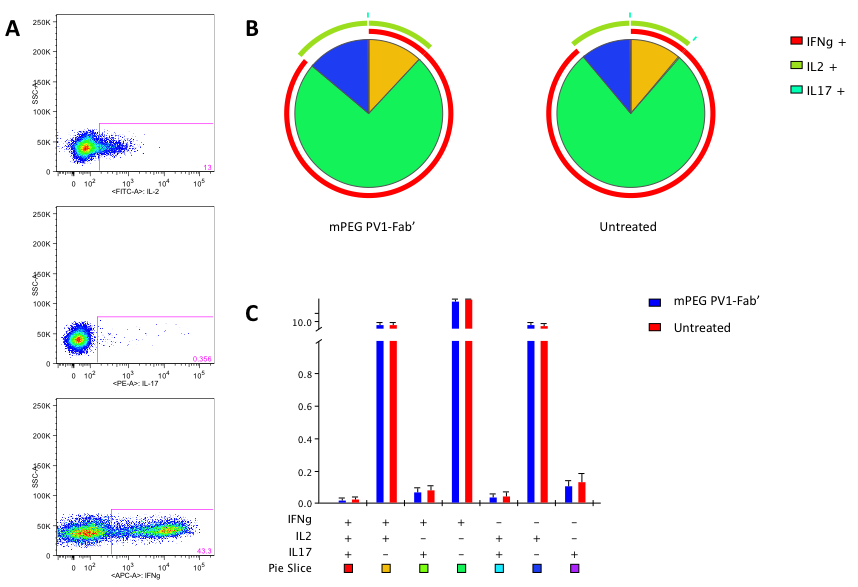

Supplement: S5 Fig — Female B10.RIII mice were immunized with 50 μg/animal of 161–180 IRBP in CFA, plus 500 ng/animal of PTx boost. Starting on day 9, mice were treated ip every 4 days with PV1 (10mg/Kg) or left untreated. On day 14 mice were sacrificed and dLN were collected for immunophenotyping and evaluation of cytokine production. For the intracellular staining of IFN-γ, IL-2 and IL-17 cells were collected from dLN, plated at 1x10^6^ cells/well concentration and stimulated overnight with 100 ng/mL of PMA and 500 ng/mL of ionomycin, plus GolgiPlug at manufacturer’s recommended concentrations. (A) Representative plots showing IFN-γ, IL-17 and IL-2 production by CD3+CD8+ cells. (B) Pie charts (C) and absolute frequency of CD8+IFN-γ+ cells. (TIFF) [file pone.0171822.s005.tiff]
